# Cardiac HDAC3 Disruption Contributes to HDAC Inhibitor-Induced QT Prolongation

**DOI:** 10.3390/cells15100902

**Published:** 2026-05-14

**Authors:** Jiao Lu, Christopher Ward, Sichong Qian, Lilei Zhang, Jiang Chang, Zheng Sun

**Affiliations:** 1 Department of Medicine, Division of Endocrinology, Diabetes, and Metabolism, Baylor College of Medicine, One Baylor Plaza, R616 ABBR Bldg., Houston, TX 77030, USA; 2 Department of Integrative Physiology, Baylor College of Medicine, Houston, TX 77030, USA; christopher.ward@bcm.edu; 3 Department of Molecular and Human Genetics, Baylor College of Medicine, Houston, TX 77030, USA; 4 Institute for Biosciences and Technology, Center for Genomics and Precision Medicine, Texas A&M University, Houston, TX 77030, USA; jiangchang@tamu.edu; 5 Department of Molecular and Cellular Biology, Baylor College of Medicine, One Baylor Plaza, R616 ABBR Bldg., Houston, TX 77030, USA

**Keywords:** histone deacetylase 3, histone deacetylase inhibitors, cancer, toxicity, electrocardiogram, cardiac electrical abnormalities, QT prolongation, potassium channels

## Abstract

Histone deacetylase (HDAC) inhibitors are approved for cancer treatment and are being investigated for a wide range of other diseases. Despite their therapeutic promise, clinical studies have reported cardiac side effects, particularly electrocardiogram (EKG) abnormalities, with QT interval prolongation being one of the most consistently reported findings. The mechanisms underlying these cardiac effects remain unclear. In this study, we investigated the role of HDAC3 in cardiac electrophysiology. We found that postnatal depletion of cardiac HDAC3 in mice caused QT interval prolongation, recapitulating the EKG abnormalities reported with HDAC inhibitor use. Adult-onset inducible depletion of cardiac HDAC3 induced additional EKG abnormalities, including T-wave flattening, inversion, and biphasic T waves, which are also observed clinically. Loss of HDAC3 deacetylase activity, without affecting HDAC3 protein levels, was sufficient to induce QT prolongation. Disruption of HDAC3 function altered the expression of ion channel genes, including the downregulation of potassium channel genes such as *Kcnh2*, *Kcne1*, and *Kcnip2*. Moreover, a single dose of HDAC inhibitors, romidepsin or mocetinostat, caused reversible QT prolongation in mice. Consistent with these findings, HDAC inhibitor treatment altered the expression of potassium channel genes, with a predominant downregulation of multiple Kcn family members, including *Kcnq1*, *Kcnh2*, and *Kcnip2*. These findings establish HDAC3 enzymatic activity as a key regulator of cardiac repolarization and provide mechanistic insight into HDAC inhibitor-associated cardiotoxicity.

## 1. Introduction

Histone deacetylases (HDACs) remove acetyl groups from lysine residues on both histone and non-histone proteins, thereby regulating chromatin structure and protein function. Based on their catalytic cofactors, HDACs are classified as either zinc-dependent or NAD^+^-dependent enzymes. The classical zinc-dependent HDACs are further divided into class I, class II (subdivided into IIa and IIb), and class IV according to sequence homology [1]. Class I HDACs include HDAC1, 2, 3, and 8; class IIa includes HDAC4, 5, 7, and 9; class IIb includes HDAC6 and HDAC10; and HDAC11 is the sole member of class IV. In contrast, the NAD^+^-dependent class III HDACs, known as sirtuins, possess distinct catalytic mechanisms.

HDAC inhibitors are a class of epigenetic agents that block HDAC activity, preventing the removal of acetyl groups from lysine residues on histone and non-histone proteins and thereby modulating gene expression. Several HDAC inhibitors, including vorinostat, romidepsin, belinostat, and panobinostat, have been approved by the FDA to treat cancers [2]. Some HDAC inhibitors are also under investigation for the treatment of neurodegenerative diseases, inflammatory disorders, and even cardiovascular diseases [3,4,5].

However, HDAC inhibitors are reported to have cardiac toxicity, particularly EKG abnormalities. A study reported an average increase of 5.0 ms in corrected QT (QTc) interval after romidepsin infusion [6]. Romidepsin treatment also induced other EKG changes, including T-wave flattening, T-wave inversion, biphasic T waves, and minor ST-segment depression (Table 1). An average of 14.4 ms increase in QTc interval, T-wave flattening, and ST-segment depression by romidepsin was observed in another trial [7]. Notably, one study reported that a sudden death occurred within 24 h after the fifth injection of romidepsin, which was possibly attributed to fatal ventricular arrhythmia [8]. Prolonged QTc and ventricular tachycardia were also observed in some patients in this trial. Due to these cardiac toxicities, this trial was prematurely terminated. Panobinostat administration caused QTc above 500 ms and average QTc change above 60 ms from baseline in 5.6% and 9.3% of patients, respectively [9]. The changes in QT prolongation were dose-dependent. Similarly, QT prolongation was observed in vorinostat (zolinza) treatment. Prolonged QT was reported in 3.5–6.0% of cases associated with vorinostat, with a greater risk of torsade de pointes in females and older patients [10]. Moreover, in a clinical trial of belinostat, 35% of patients experienced QTc prolongation. Two patients had electrolyte abnormalities, which could have contributed to this EKG abnormality [11].

These EKG changes reflect a decrease in the rate of ventricular repolarization. A study in canines suggests that the prolonged cardiac repolarization induced by HDAC inhibitors may be mediated by altered expression of genes involved in ion channel trafficking [13]. Trichostatin A (TSA), vorinostat, or romidepsin decreased the density of cardiac sodium currents I_Na_, reduced Na_v_1.5 protein levels, but did not affect late I_Na_ amplitude [14]. An in vitro study using HL-1 atrial cardiomyocytes showed that HDAC inhibition induced action potential prolongation and potassium channel remodeling [15]. Furthermore, ion channel expression and function are essential in action potential generation, in which potassium channels play a key role in cardiac repolarization. Inhibition of HDAC function could interfere with the expression of ion channels and thereby disrupt the action potential process, manifesting as EKG abnormalities.

HDAC3 is a class I histone deacetylase with robust enzymatic activity and is also required for the deacetylase function of class IIa HDACs. It plays a critical role in the regulation of cardiac function [16,17,18]. Pharmacological studies have shown that inhibition of HDAC3 can ameliorate myocardial ischemia–reperfusion injury, reduce cardiomyocyte hypertrophy and heart failure induced by transverse aortic constriction, and mitigate metabolic and inflammatory alterations associated with heart failure [19,20,21].

Genetic mouse models have further defined the role of HDAC3 in the heart [16,17,18]. Early developmental deletion of HDAC3 is deleterious, leading to cardiac hypertrophy, increased myocardial triglyceride accumulation, and fatal heart failure [16]. In contrast, postnatal deletion of HDAC3 enhances ejection fraction under a normal chow diet but results in lethal heart failure with reduced ejection fraction under high-fat diet or pressure overload stress [17,18]. Collectively, these findings highlight a central role for HDAC3 in the regulation of cardiac hypertrophy and lipid metabolism. Its function is also essential during heart development. Deletion of HDAC3 in the second heart field results in embryonic lethality, accompanied by severe structural abnormalities such as ascending aortic dilation, double outlet right ventricle, and defective valve formation [22]. Similarly, loss of HDAC3 in the developing epicardium leads to ventricular myocardial hypoplasia and a reduction in epicardium-derived cells, indicating that HDAC3 is required for proper myocardial growth during development [23].

Despite the established roles of HDAC3 in cardiac contraction and development, its function in cardiac electrophysiology remains largely unexplored. It is also unclear how HDAC inhibitors alter EKG parameters in preclinical rodent models in ways that inform human studies. Moreover, the mechanisms underlying the electrical abnormalities observed in clinical trials of HDAC inhibitor-based cancer therapies remain poorly defined.

In this study, we demonstrate that disruption of HDAC3 recapitulates EKG abnormalities associated with the clinical use of HDAC inhibitors. We further identify a transcriptional program involving ion channel genes that is altered by HDAC3 dysfunction. These findings provide mechanistic insight into the cardiotoxic effects of HDAC inhibitors, particularly in patients at increased risk for cardiac electrical abnormalities.

## 2. Methods

### 2.1. Animals

All animal care and procedures were approved by the Institutional Animal Care and Use Committee (IACUC) at Baylor College of Medicine. HDAC3^fl/fl^/MCK-Cre (KO) mice, HDAC3^fl/fl^/αMHC-MerCreMer (iKO) mice, NS-DADm (KI) mice, and αMHC-MerCreMer (MCM) mice were previously described [17,18,24,25,26]. All mice were on the C57BL/6 genetic background. Mice were maintained on a 12 h light/dark cycle and had unrestricted access to drinking water and chow diet (LabDiet, St. Louis, MO, USA; PicoLab 5V5R). Male and female mice aged 4 to 12 months were used for all experiments unless otherwise indicated. Tamoxifen (Sigma-Aldrich, St. Louis, MO, USA; T2859) was dissolved in corn oil (Sigma-Aldrich, St. Louis, MO, USA; C8267) at the concentration of 10 mg/mL and injected intraperitoneally at 20 mg/kg per day for 4 consecutive days. Romidepsin (MedChemExpress, Monmouth Junction, NJ, USA; HY-15149) was diluted in phosphate-buffered saline (Corning Life Sciences, Corning, NY, USA; 21-031-CV) at a concentration of 0.5 mM and injected intraperitoneally at 1 mg/kg. Mocetinostat (MedChemExpress, Monmouth Junction, NJ, USA; HY-12164) was diluted in PEG 400 (Lab Alley, Austin, TX, USA; PEG400F) at a concentration of 4 mg/mL and injected intraperitoneally at 10 mg/kg.

### 2.2. Surface EKG

For recording the surface EKG signals in mice, anesthesia was induced with 3% isoflurane and confirmed by the lack of response to firm pressure on one of the hind paws. During EKG recording, isoflurane was reduced to 2% and adjusted to maintain a heart rate in the range of 450–500 beats per minute. Body temperature was maintained at 36.5–37.5 °C. Mice were placed in a supine position. EKG paste was applied to the paws of the mice, which were then connected to the platform’s electrode pads and secured with skin tape. Signals were collected using Rodent Surgical Monitor+ (Indus Instruments, Webster, TX, USA). Data were recorded and analyzed using LabChart 8 software (ADInstruments, Dunedin, New Zealand). Heart rate (HR), RR interval, PR interval, QRS duration, and QT interval were calculated. Corrected QT (QTc) was calculated using Mitchell’s formula [27].

### 2.3. RNA Sequencing

Total RNA was extracted using the RNeasy Mini Kit (Qiagen, Hilden, Germany; 74106). Bulk RNA sequencing was performed using total RNA. Sequencing libraries were run on the BGI MGISEQ-2000 platform to an average depth of 60 million reads per sample. The sequencing data were filtered with SOAPnuke by removing reads containing the sequencing adapter. The resulting clean reads were obtained and stored in FASTQ format and mapped to the reference genome GRCm38.p6 using HISAT2. Bowtie2 was applied to align the clean reads to the reference coding gene set, and gene expression levels were calculated with RSEM. Differential expression analysis was performed using DESeq2. Genes were considered differentially expressed if the adjusted *p*-value (Benjamini–Hochberg method) was <0.05. Functional annotation analysis was performed using DAVID Bioinformatics Resources. Enrichment analysis for biological processes and pathways was conducted using Gene Ontology (GO) and KEGG.

### 2.4. Statistics

Differences in EKG outcome measures were analyzed using a two-tailed unpaired Student’s *t*-test for experiments comparing two different genotype groups, and a paired *t*-test for experiments on the same subjects before and after tamoxifen or HDAC inhibitor treatment. A value of *p* < 0.05 was considered statistically significant. Statistical analyses were conducted using SPSS Statistics 19. Estimated sample sizes were determined based on previous publications and our experience to obtain statistically significant results. The sample size for each group is indicated in the figures or figure legends. Experimental mice were randomly assigned to experimental or control groups. Investigators were blinded to the genotypes of individual animals during experiments and result assessment. Differential RNA-seq genes were identified by adjusting *p* values for multiple testing using an FDR (Benjamini–Hochberg method) threshold of <0.05.

## 3. Results

**Figure 1:**
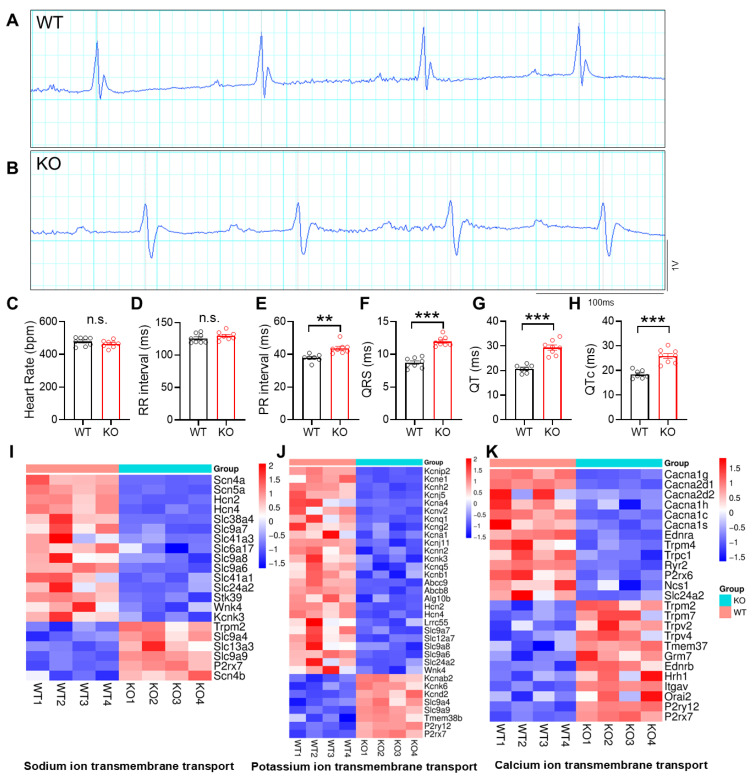
EKG abnormalities in mice depleted of cardiac HDAC3. (**A**,**B**) Representative image of surface EKG tracings of WT and KO. (**C**) Heart rate during surface EKG recording, *n* = 7–8 per group. (**D**) RR interval *n* = 7–8 per group. (**E**) PR interval. *n* = 7–8 per group. (**F**) QRS duration, *n* = 7–8 per group. (**G**) QT duration, *n* = 7–8 per group. (**H**) QTc duration, *n* = 7–8 per group. (**I**–**K**) Sodium, potassium and calcium ion transmembrane transports were among the top enriched biological processes (BP) from Gene Ontology analysis for pooled DEGs (KO vs. WT, q < 0.05). Each DEG in the sodium, potassium or calcium ion transmembrane transport process from the analysis was listed. Data are mean ± SEM. ** *p* < 0.01, *** *p* < 0.001, n.s. indicates not significant. Data were obtained from combined female and male mice aged 3–4 months (**A**–**H**) or 6-week-old male mice (**I**–**K**). WT, HDAC3^fl/fl^ mouse. KO, HDAC3^fl/fl^:MCK-Cre mouse.

HDAC3 belongs to class I HDACs, and the enzymatic activity of class II HDACs depends on HDAC3 [28]. To examine the role of HDAC3 in cardiac electrical activity, we analyzed a mouse model with cardiac-specific deletion of HDAC3 using the MCK-Cre line (KO mice). Compared with wild-type (WT) controls, KO mice exhibited increased PR and QRS duration on surface EKG analysis at 4 months of age (Figure 1A–F). KO mice also showed a significant prolongation of the QT interval (Figure 1G,H), a phenotype commonly observed with clinical use of HDAC inhibitors.

RNA sequencing of HDAC3 KO hearts from 6-week-old mice revealed changes in genes involved in ion transport (Figure 1I–K), including sodium, potassium, and calcium ion channels and transporters. Sodium and calcium channel genes were altered in both directions following HDAC3 depletion. Notably, most potassium channel genes were downregulated, including Kcnh2, Kcnip2, and Kcne1. We further validated the reduced expression of potassium channel genes by RT–qPCR (Supplementary Materials Figure S1). The bidirectional changes observed in sodium channel genes may reflect their complex roles in cardiac excitability, as both increased and decreased sodium channel activity have been associated with arrhythmogenic phenotypes [29,30]. This may explain why their expression changes are not uniformly directional, in contrast to potassium channel genes. We used 6-week-old mice to capture early transcriptional changes, thereby minimizing secondary alterations associated with disease progression or long-term compensatory remodeling.

**Figure 2:**
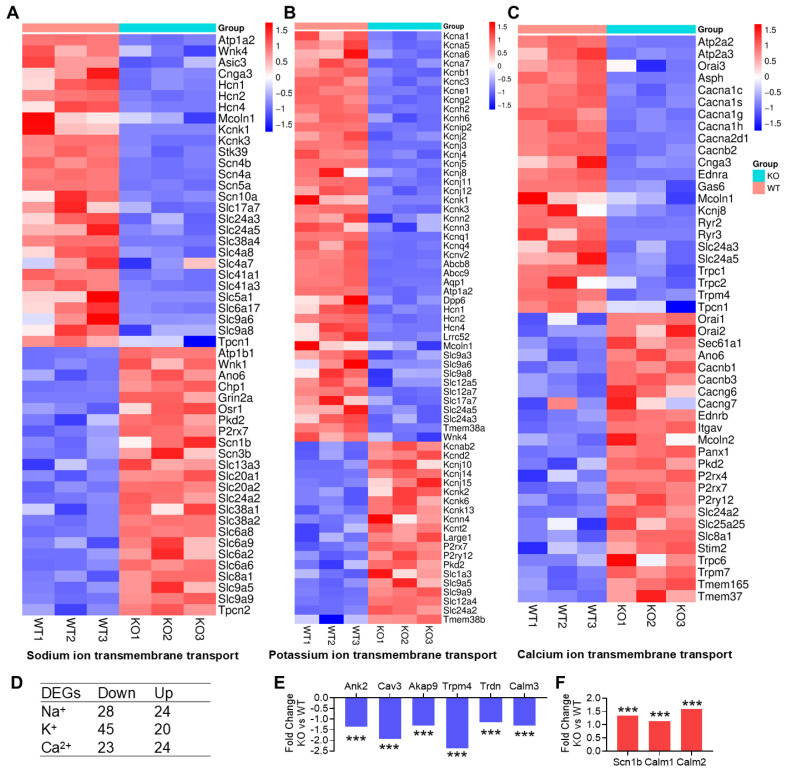
Transcriptomic profile of HDAC3 knockout hearts on ion transport. (**A**–**C**) Sodium, potassium and calcium ion transmembrane transports were among the top enriched biological processes (BP) from Gene Ontology analysis for pooled DEGs (KO vs. WT, q < 0.05). Each DEG in the sodium, potassium or calcium ion transmembrane transport process from the analysis was listed. (**D**) Summary of gene count of downregulated genes and upregulated genes in panel (**A**–**C**). (**E**) Example of other downregulated genes important for EKG not listed in panel (**A**–**C**). (**F**) Example of other upregulated genes important for EKG not listed in panel (**A**–**C**). Data are mean ± SEM. *** *p* < 0.001. Data were obtained from male mice aged ~3 months. WT, HDAC3^fl/fl^ mouse. KO, HDAC3^fl/fl^:MCK-Cre mouse.

In KO hearts of mice older than 3 months, the changes in ion channel expression were more prominent than those at 6 weeks of age (Figure 2A–C). Again, a similar number of genes were upregulated and downregulated in sodium or calcium transport (Figure 2D). However, among genes involved in potassium transport, most were downregulated rather than upregulated (45 versus 20). More strikingly, there was an overall decrease in the Kcn gene family. Several of these genes are associated with long QT syndrome, as mutations in those genes are known to prolong QT intervals [31]. Among the downregulated genes are those that do not directly fall into the ion transport category but are important for regulating the QT interval, e.g., *Ank2*, *Cav3*, *Akap9*, *Trpm4*, *Trdn*, and *Calm3* (Figure 2E). Other genes including *Scn1b*, *Calm1*, and *Calm2* were upregulated (Figure 2F).

For the genes related to each subtype of genetic long QT syndrome, we found that 11 out of 13 genes were reduced by HDAC3 depletion (Table 2). These include *Kcnq1*, *Kcnh2*, *Kcne1*, *Kcnj2*, and *Kcnj5* in the potassium channel family, responsible for long QT syndrome types 1, 2, 5, 7, and 13; *Scn5a* and *Scn4b* in the sodium channel family, responsible for long QT syndrome types 3 and 10; and *Cacna1c* in the calcium channel family, responsible for long QT syndrome type 8, as well as *Ank2*, *Cav3*, and *Akap9*, which are associated with long QT syndrome types 4, 9, and 11. These results support the idea that the EKG abnormalities in HDAC3-depleted hearts could be due to changes in ion transport.

**Figure 3:**
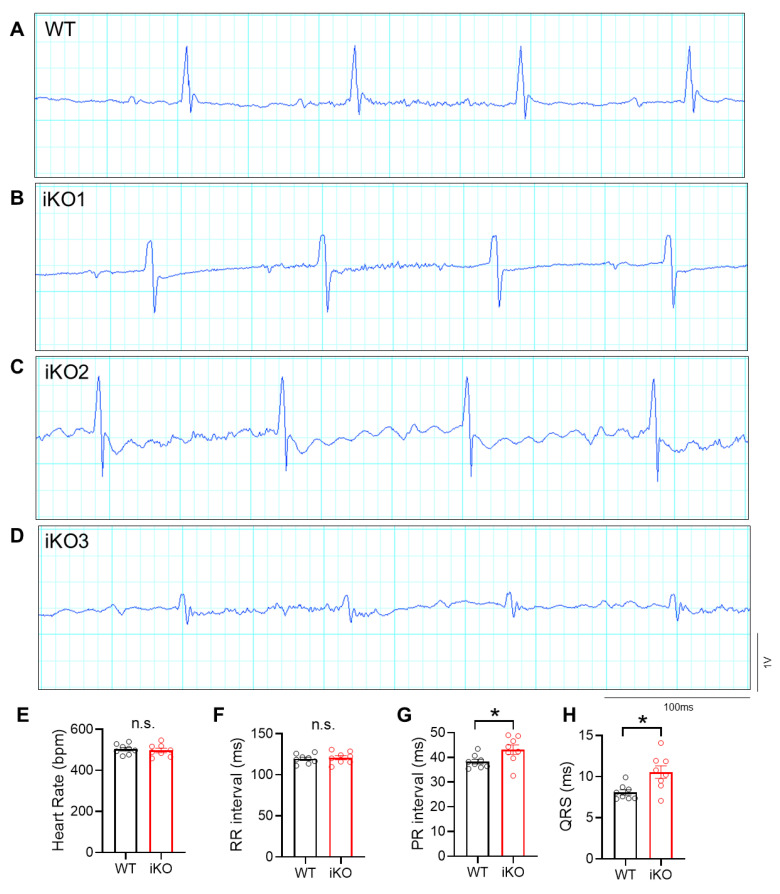
EKG abnormalities in adult mouse hearts with inducible depletion of HDAC3. (**A**–**D**) Representative image of surface EKG tracings of WT and iKO. (**E**) Heart rate during surface EKG recording, *n* = 8 per group. (**F**) RR interval *n* = 8 per group. (**G**) PR interval. *n* = 8 per group. (**H**) QRS duration, *n* = 8 per group. Data are mean ± SEM. * *p* < 0.05, n.s. indicates not significant. Data were from combined female and male mice. WT, HDAC3^fl/fl^ mouse. iKO, HDAC3^fl/fl^:Mer-Cre-Mer mouse. Both WT and iKO were injected with tamoxifen at 4 months of age. EKG recordings were performed 2 months later.

To examine the development-independent function of HDAC3, we next performed surface EKG in mice with adult-onset inducible knockout of HDAC3 using the aMHC-MerCreMer line (iKO mice). We also found a significant increase in PR interval and QRS duration in 6-month-old mice after tamoxifen injection at 4 months of age, compared to control HDAC3^fl/fl^ mice (Figure 3A–H). These changes were similar to those observed in the constitutive KO mouse line. In addition, morphological changes in the T wave were observed in all iKO mice. We observed T-wave flattening in half of the iKO mice (4 out of 8; Figure 3B). A total of 2 out of 8 mice showed T-wave inversion (Figure 3C), and 2 showed biphasic T waves (Figure 3D). Such T-wave changes are consistent with those reported in clinical trials of HDAC inhibitors [6,7]. Due to the abnormal T-wave morphology, the QT interval was not quantifiable in these mice. As an additional control group, we also injected the MerCreMer mouse line with tamoxifen using the same method. There was no change in EKG tracings compared to baseline (Supplementary Materials Figure S2). This result confirms that the EKG abnormalities in iKO mice were caused by HDAC3 depletion rather than the toxicity of tamoxifen or the Cre allele.

**Figure 4:**
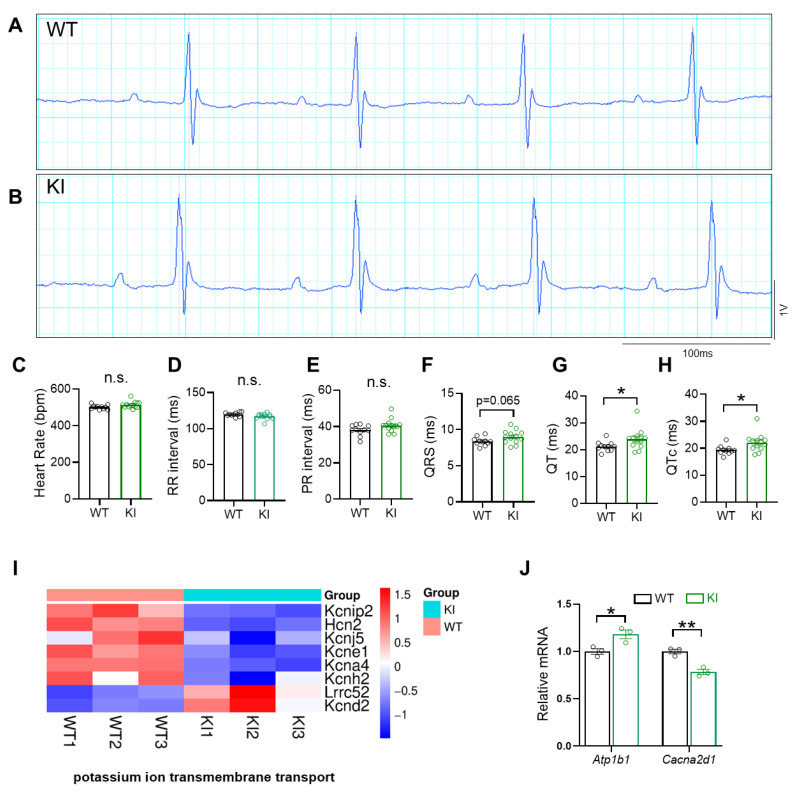
EKG abnormalities in knock-in mice without HDAC3 deacetylase activity. (**A**,**B**) Representative image of surface EKG tracings of WT and KI. (**C**) Heart rate during surface EKG recording, *n* = 11–13 per group. (**D**) RR interval *n* = 11–13 per group. (**E**) PR interval. *n* = 11–13 per group. (**F**) QRS duration, *n* = 11–13 per group. (**G**) QT duration, *n* = 11–13 per group. (**H**) QTc duration, *n* = 11–13 per group. (**I**) Potassium ion transmembrane transport was among the top enriched biological processes (BP) from Gene Ontology analysis for pooled DEGs (KI vs. WT, q < 0.05). Each DEG in the potassium ion transmembrane transport process from the analysis was listed. (**J**) Relative mRNA expression of *Atp1b1*, a gene involved in sodium ion transmembrane transport, and *Cacna2d1*, a gene involved in calcium ion transmembrane transport, differed between WT and KI mice. Data are mean ± SEM. * *p* < 0.05, ** *p* < 0.01, n.s. indicates not significant. Data were obtained from combined female and male mice aged 2–3 months (**A**–**H**) or 6-week-old male mice (**I**,**J**). WT, wild-type C57BL/6 mouse. KI, NS-DADm mouse.

Since HDAC3 can have enzyme-independent functions, we further studied whether loss of HDAC3 deacetylase catalytic activity is sufficient to induce EKG changes. We used the NS-DAD mouse line, which has normal HDAC3 expression but lacks deacetylase function [25]. This is due to mutations in the Deacetylase-Activating Domain (DAD) of the corepressors N-CoR and SMRT. EKG analysis revealed QT prolongation in NS-DADm mice compared to WT controls (Figure 4A–H). The PR interval and QRS duration were not changed. Our transcriptomic analysis showed that potassium ion transmembrane transport activity was impacted by loss of HDAC3 deacetylase activity (Figure 4I). However, sodium and calcium ion transmembrane transport were not affected, except for increased expression of *Atp1b1* and *Cacna2d1* (Figure 4J).

Both KO and iKO mouse lines showed prolonged PR interval and QRS duration compared to WT controls, while these changes were absent in the NS-DADm mouse line. These results suggest that HDAC3 deacetylase activity is not required for early phases of cardiac electrical activity but instead primarily affects cardiac repolarization. This is supported by the overall downregulation of genes involved in potassium transport in NS-DADm mice, with little change in genes involved in sodium or calcium transport. In contrast, KO hearts showed changes in genes involved in sodium, calcium, and potassium ion transport.

**Figure 5:**
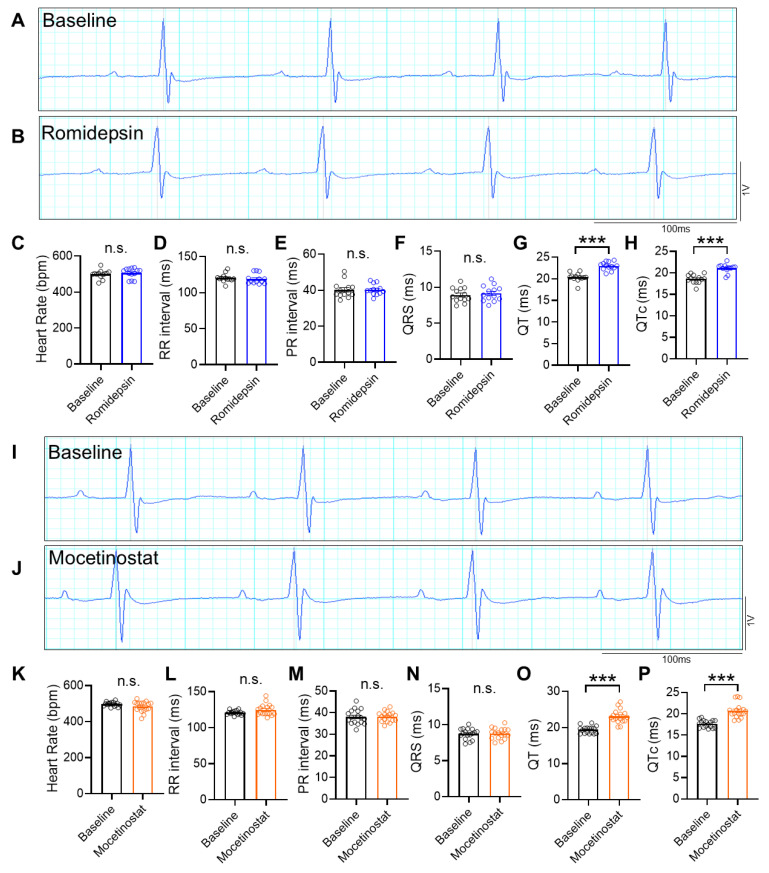
EKG abnormalities in WT mice administered with HDAC inhibitors. (**A**,**B**) Representative image of surface EKG tracings of a WT mouse before (baseline) and 17 h after romidepsin administration at 1 mg/kg intraperitoneally. (**C**) Heart rate during surface EKG recording, *n* = 13 per group. (**D**) RR interval *n* = 13 per group. (**E**) PR interval. *n* = 13 per group. (**F**) QRS duration, *n* = 13 per group. (**G**) QT duration, *n* = 13 per group. (**H**) QTc duration, *n* = 13 per group. (**I**,**J**) Representative image of surface EKG tracings of a WT mouse before and 17 h after mocetinostat administration at 10 mg/kg intraperitoneally. (**K**) Heart rate during surface EKG recording, *n* = 16 per group. (**L**) RR interval *n* = 16 per group. (**M**) PR interval. *n* = 16 per group. (**N**) QRS duration, *n* = 16 per group. (**O**) QT duration, *n* = 16 per group. (**P**) QTc duration, *n* = 16 per group. *** *p* < 0.001, n.s. indicates not significant. Data were obtained from combined female and male mice aged 2–4 months. WT, wild-type C57BL/6 mouse.

Lastly, we wondered whether HDAC inhibitors could cause EKG abnormalities in healthy subjects. We first tested romidepsin, a potent and selective class I HDAC inhibitor. We measured the baseline EKG of WT mice before treatment. We then injected them with 1 mg/kg romidepsin intraperitoneally as a single dose and recorded the EKG the next day. We used this dose because it has been used in studies of cancer treatment in mice [32,33]. EKG was measured 17 h after injection, based on a study that observed EKG changes at 17 h after HDAC inhibitor treatment in canines [13]. We found that the QT interval was significantly increased after romidepsin administration (Figure 5A–H). There was a 2.6 ms increase compared with baseline before treatment. The PR interval and QRS duration were not changed.

Similarly, administration of mocetinostat (MGCD0103, a class I HDAC inhibitor) at 10 mg/kg [34] as a single intraperitoneal dose in WT mice also led to QT prolongation (Figure 5I–P). It caused a 3.6 ms increase in the QT interval without changing the PR interval or QRS duration. These results suggest that HDAC inhibitors interfere with cardiac repolarization. The drug effects appear within 20 h.

**Figure 6:**
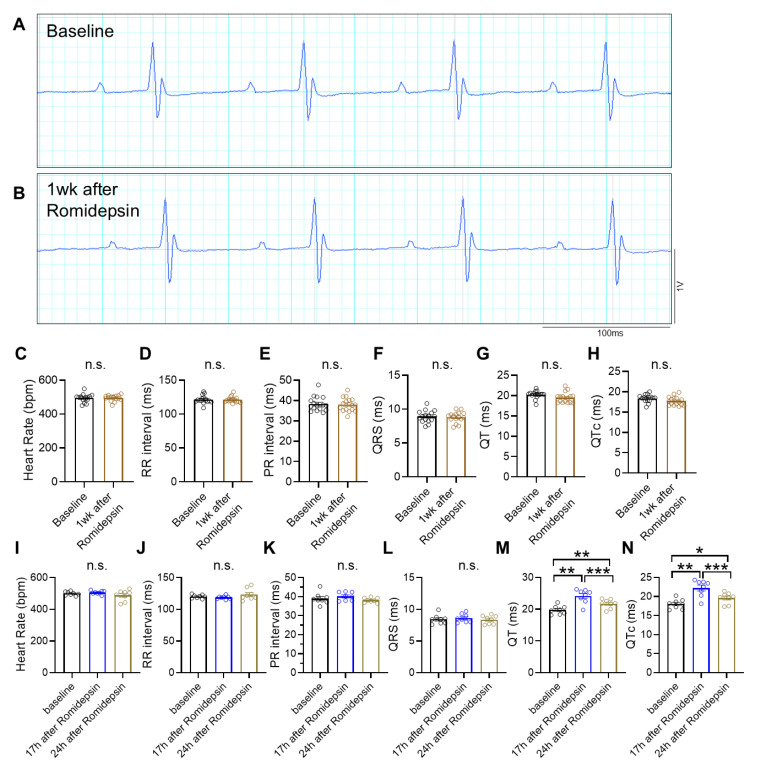
EKG tracings of WT mice at 1 week or 24 h following romidepsin administration. (**A**,**B**) Representative image of surface EKG tracings of a WT mouse before (baseline) and 1 week after romidepsin administration at 1 mg/kg intraperitoneally. (**C**) Heart rate during surface EKG recording, *n* = 16 per group. (**D**) RR interval, *n* = 16 per group. (**E**) PR interval, *n* = 16 per group. (**F**) QRS duration, *n* = 16 per group. (**G**) QT duration, *n* = 16 per group. (**H**) QTc duration, *n* = 16 per group. (**I**–**N**) Heart rate, RR interval, PR interval, QRS duration, QT duration, and QTc duration of WT mice before (baseline), 17 h after, and 24 h after romidepsin administration at 1 mg/kg intraperitoneally, *n* = 8 per group. * *p* < 0.05, ** *p* < 0.01, *** *p* < 0.001, n.s. indicates not significant. Data were obtained from combined female and male mice aged 2–4 months. WT, wild-type C57BL/6 mouse.

The EKG changes induced by HDAC inhibitors are reversible. When EKG tracings were recorded 1 week after romidepsin injection, the QT interval returned to baseline levels (Figure 6A–H). Further temporal analysis revealed that, at 24 h post-injection, the QT interval was significantly shorter than at 17 h post-injection (Figure 6I–N). The decline in QT prolongation after 24 h likely reflects the clearance of the drug from the body.

We next examined the expression of HDACs following treatment with romidepsin or mocetinostat. Romidepsin, a class I HDAC inhibitor, did not alter the expression of class I HDACs but decreased the expression of *Hdac7* and *Hdac9* (class IIa) and increased *Hdac6* expression (class IIb) (Supplementary Materials Figure S3). In contrast, another class I HDAC inhibitor, mocetinostat, decreased the expression of *Hdac2* and *Hdac8* (class I), *Hdac10* (class IIb), and *Hdac11* (class IV), while increasing *Hdac4* and *Hdac7* (class IIa) (Supplementary Materials Figure S4). Despite these distinct expression profiles, both drugs induced similar EKG changes, suggesting that class I HDAC inhibitors do not necessarily alter the expression of class I HDACs to exert their effects. Instead, inhibition of class I HDAC enzymatic activity may represent the shared mechanism. Consistent with this, in NS-DADm mice [25], disruption of HDAC3 enzymatic activity without changes in HDAC3 expression was sufficient to induce QT prolongation (Figure 4A–H). These findings highlight the importance of HDAC enzymatic activity in regulating cardiac electrical function.

**Figure 7:**
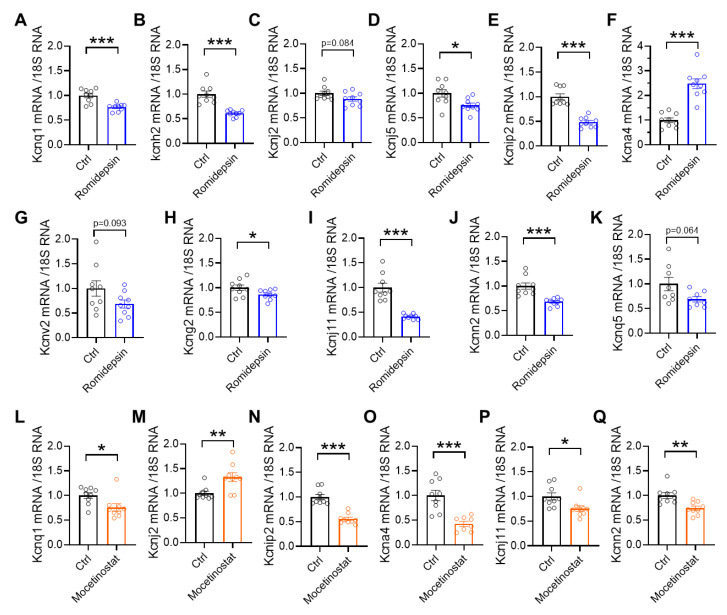
Expression of cardiac KCN genes in mice administered HDAC inhibitors. (**A**–**K**) Quantitative PCR analysis of cardiac mRNA expression for the indicated genes in wild-type C57BL/6 mice treated with vehicle (Ctrl) or romidepsin (1 mg/kg, intraperitoneally), *n* = 8–9 per group. (**L**–**Q**) Quantitative PCR analysis of cardiac mRNA expression for the indicated genes in wild-type C57BL/6 mice treated with vehicle (Ctrl) or mocetinostat (10 mg/kg, intraperitoneally), *n* = 9 per group. Mouse hearts were harvested 6 h after romidepsin or mocetinostat injection. mRNA levels were normalized to 18S rRNA. Data are mean ± SEM. * *p* < 0.05, ** *p* < 0.01, *** *p* < 0.001. Data were obtained from combined female and male mice aged 2–4 months.

In HDAC3-disrupted mouse models, we observed an overall reduction in potassium channel gene expression. We therefore asked whether HDAC inhibitors induce similar changes that could contribute to QT prolongation. To test this, we examined the Kcn family genes identified in HDAC3 KO mice (Figure 1J). Romidepsin treatment significantly decreased the expression of *Kcnq1*, *Kcnh2*, *Kcnj5*, *Kcnip2*, *Kcng2*, *Kcnj11*, and *Kcnn2*, while increasing *Kcna4* (Figure 7A–K). Mocetinostat treatment decreased the expression of *Kcnq1*, *Kcnip2*, *Kcna4*, *Kcnj11*, and *Kcnn2*, while increasing *Kcnj2* expression (Figure 7L–Q).

In summary, these findings indicate that cardiac HDAC3 disruption contributes to HDAC inhibitor-induced prolongation of cardiac repolarization.

## 4. Discussion

Our study revealed that HDAC3 depletion or inhibition in mouse models led to EKG abnormalities. We were able to recapitulate the clinical observations of QT prolongation and T-wave morphological changes in patients under treatment with HDAC inhibitors for cancer. Our results bring attention to the cardiac toxicity of cancer therapies that target HDACs, especially in patients at higher risk of cardiac arrhythmia. We showed that the transcriptional profile of ion channel transport was significantly altered by HDAC3 dysfunction. The expression levels of multiple key potassium channels decreased dramatically. Most of the genes associated with genetic long QT syndrome were reduced. Thus, HDAC3 regulates the expression of key ion channels related to QT prolongation. These changes likely affect the repolarization process during action potential generation and therefore increase cardiac relaxation time, as evidenced by a prolonged QT interval.

Given the central role of potassium channels in cardiac repolarization, the enrichment of potassium channel gene changes observed in our dataset is biologically consistent with the QT prolongation phenotype. Cardiac repolarization is predominantly governed by outward K^+^ currents, and reductions in the expression or function of these channels can lead to prolonged action potential duration and QT interval [35]. In contrast, sodium and calcium channels primarily regulate depolarization and plateau phases [36], which may explain why their transcriptional changes were more variable and less uniformly directional. Thus, the predominant downregulation of potassium channel genes likely reflects their key role in repolarization and the sensitivity of these pathways to transcriptional perturbation following HDAC3 disruption.

Mechanistically, HDAC3 is a transcriptional regulator that functions within multiprotein corepressor complexes to modulate chromatin accessibility through deacetylation of histone and non-histone proteins. Loss of HDAC3 activity is therefore expected to alter gene expression programs. In our study, disruption of HDAC3 led to coordinated downregulation of multiple potassium channel genes, suggesting a transcriptional mechanism. While we did not directly assess acetylation levels at specific gene loci, HDAC3 may regulate potassium channel expression through chromatin remodeling at their regulatory regions or through modulation of transcription factors that control ion channel gene programs. Additionally, because HDAC3 is required for the enzymatic activity of class IIa HDACs, its disruption may have broader effects on transcriptional networks governing cardiac electrophysiology. Future studies examining locus-specific histone acetylation, HDAC3 genomic occupancy, and potential non-histone substrates will be important to define the precise mechanisms linking HDAC3 activity to potassium channel regulation.

Not only were ion channels directly affected by HDAC3 KO, but other genes critical for protein trafficking and localization to cellular membranes were also altered. Ankyrin-B is an important scaffolding protein that organizes and anchors proteins at the cell membrane, particularly ion channels and transporters. The gene *Ank2*, which encodes ankyrin-B, was decreased following HDAC3 depletion (Figure 2E). Similarly, *Cav3*, which encodes caveolin-3, was downregulated. Caveolin-3 is a key structural component of caveolae, specialized membrane microdomains. Another gene decreased by HDAC3 KO, *Akap9*, encodes a scaffolding protein involved in maintaining the structure of the Golgi apparatus and centrosomes, as well as other cellular processes. In contrast, we did not observe significant changes in the expression of major gap junction genes, including Gja1 (Cx43), suggesting that gap junction remodeling is unlikely to be a primary contributor to the observed electrical abnormalities.

In addition to HDAC3, other HDACs are also implicated in the regulation of cardiac electrical activity. For example, cardiac-specific deletion of both HDAC1 and HDAC2 (αMHC-Cre) resulted in severe arrhythmias by postnatal day 10, accompanied by dilated cardiomyopathy and lethality by day 14 [37]. In a transverse aortic constriction (TAC) model, HDAC2 expression was upregulated, which was associated with increased susceptibility to ventricular arrhythmias, reduced transient outward K^+^ current (I_to,f), and prolonged action potential duration in cardiomyocytes [37]. Knockdown of HDAC2 by RNA interference in this model reversed these phenotypes, reducing arrhythmia susceptibility, restoring I_to,f, and shortening action potential duration.

Compared with HDAC1/2, HDAC3 appears to play a distinct role in cardiac electrophysiology. HDAC3 possesses robust deacetylase activity and is required for the enzymatic function of class IIa HDACs, placing it at a central regulatory position within the HDAC network. In our study, disruption of HDAC3 alone was sufficient to induce QT prolongation and broad transcriptional changes in ion channel genes, particularly the downregulation of potassium channel genes that are critical for cardiac repolarization. This phenotype closely mirrors the EKG abnormalities observed with HDAC inhibitor treatment, supporting a prominent role for HDAC3 in maintaining normal cardiac electrical function.

Having said this, it is unlikely that HDAC3 acts in isolation. HDACs function within multiprotein complexes and often exhibit overlapping or compensatory roles. Class I HDACs share structural similarities and may regulate common transcriptional programs, whereas class II HDACs can modulate signaling pathways that indirectly affect ion channel expression and function. Therefore, other HDACs may also influence cardiac electrophysiology, either independently or through interaction with HDAC3-containing complexes. Future studies are needed to delineate the specific contributions of individual HDAC isoforms and to determine whether their effects converge on shared pathways governing cardiac repolarization and excitability. In this context, different HDAC isoforms may exert distinct and sometimes opposing effects on cardiac function.

In addition to class I HDACs, class IIb HDACs such as HDAC6 have also been implicated in cardiac disease, particularly in heart failure with preserved ejection fraction (HFpEF), where HDAC6 inhibition has been reported to exert cardioprotective effects [38]. This contrast likely reflects isoform-specific functions. Unlike HDAC3, which primarily regulates transcription in the nucleus, HDAC6 is predominantly cytoplasmic and modulates protein trafficking and cellular stress responses [39]. Notably, HDAC3 expression is dynamically regulated in disease states, with upregulation reported in ischemia–reperfusion injury and pressure overload [19,20,21], and reduced expression observed in failing human hearts [21], suggesting a dosage-sensitive role in cardiac homeostasis. However, its role in arrhythmogenic conditions remains poorly defined. In this context, the EKG abnormalities observed in our study likely reflect transcriptional dysregulation of ion channel networks, whereas the beneficial effects of HDAC inhibition in other models may arise from isoform-specific targeting or actions in non-cardiomyocyte cell types. These distinctions highlight the isoform-specific and context-dependent roles of HDACs in the heart and underscore the importance of selective targeting when considering therapeutic strategies.

In our EKG study using HDAC inhibitors, we tested romidepsin and mocetinostat. We found that both drugs caused an increase in the QT interval. Notably, heart rate and RR interval were not significantly altered in our study, which may reflect the use of anesthetized conditions during EKG recordings in mice, potentially masking subtle changes in heart rate dynamics. Combined with clinical observations (Table 1) of QT prolongation, it appears that this cardiac side effect of HDAC inhibitors is widely observed in both humans and animals. Sudden cardiac death is the main adverse outcome of long QT syndrome and is due to a potentially fatal ventricular arrhythmia, torsades de pointes (TdP) [40]. Other adverse outcomes of long QT syndrome include syncope, seizures, and ventricular fibrillation [41]. Our study highlights this potential risk and draws attention to the drug safety of HDAC inhibitors. Importantly, our observation that HDAC inhibitors induce QT prolongation in healthy mice suggests that these effects are not restricted to cancer-specific contexts and may reflect direct cardiac actions independent of disease-associated immune or inflammatory alterations. We suggest that HDAC inhibitors such as romidepsin should be used with caution in patients with existing or potential risk for EKG abnormalities.

Transcriptional changes were detectable as early as 6 weeks but became more pronounced in older mice, suggesting that HDAC3 disruption initiates early gene regulatory alterations that progressively accumulate over time. The modest changes at 6 weeks likely represent primary transcriptional responses, whereas the broader alterations in older mice may reflect secondary adaptations and cumulative remodeling. In contrast, the EKG abnormalities induced by HDAC inhibition were rapidly reversible. QT prolongation was significantly reduced 24 h after treatment compared with the effect at 17 h and eventually returned to baseline levels. This temporal pattern is consistent with prior observations in canine models [13] and likely reflects transient pharmacologic inhibition of HDAC activity rather than sustained structural remodeling. These findings have implications for treatment strategies. The reversibility of EKG changes suggests that cardiac electrical effects of HDAC inhibitors may be manageable with appropriate dosing and monitoring. However, the progressive nature of transcriptional alterations raises the possibility that repeated or prolonged exposure could lead to cumulative changes in ion channel expression, potentially increasing arrhythmia risk over time, particularly in susceptible individuals.

In our HDAC3 genetic models, we found that loss of deacetylase activity is sufficient to cause QT prolongation, while the expression of HDAC3 remains unchanged in the heart. This finding highlights the importance of HDAC3 deacetylase activity in cardiac electrophysiology. HDAC3 is a key member of class I HDACs with strong enzymatic activity and is responsible for the enzymatic activity of class IIa HDACs [28]. When we used two class I HDAC inhibitors, romidepsin and mocetinostat, we observed the same EKG change—QT prolongation—as seen with HDAC3 deacetylase activity ablation, without affecting the PR interval or QRS duration. It is possible that the tested HDAC inhibitors act by inhibiting HDAC3 deacetylase activity. As a future direction, it will be helpful to further examine how HDAC3 or HDAC inhibitors rewire the genome and thereby impact the expression of genes related to repolarization during action potential generation.

## Figures and Tables

**Table 1:** Clinical reports of EKG changes caused by HDAC inhibitor therapy.

HDACi	Key EKG Findings	Other EKG Changes/Notes	References
Romidepsin (FK228) 14 mg/m^2^ as a 4 h infusion	QT prolongation and ventricular arrhythmia: Among 15 patients with metastatic neuroendocrine tumors, cases of prolonged QTc and ventricular tachycardia, 1 sudden death possibly attributed to fatal ventricular arrhythmia. (study prematurely terminated due to serious cardiac toxicity)	No difference in plasma depsipeptide levels in patients with versus without cardiac adverse events.	[8]
Romidepsin (FK228) 14 mg/m^2^ as a 4 h infusion	QT prolongation and ST/T Wave Changes: Among 42 patients with cutaneous or peripheral T-cell lymphoma, a mean increase of 14.4 ms in QTcF interval	T-wave flattening or ST-segment depression observed in more than half of EKGs	[7]
Romidepsin (FK228) 14 mg/m^2^ as a 4 h infusion; analysis during day 1 exposure to drug vs. baseline	QT prolongation and ST/T Wave Changes: Among 110 patients with peripheral T-cell lymphoma and advanced cancer, a mean increase of 5.0 ms in QTcF interval, with QTcF increased in 68% of events.	T-wave flattening or inversion, biphasic T-waves, and minor ST-segment depression.	[6]
Panobinostat (LBH589) 20 mg/dose, 30 mg/dose, 40 mg/dose, or 60 mg/dose weekly	QT prolongation: At 60 mg/dose, among 54 patients with hematologic malignancies and solid tumors (average age of 60 years, range 16–88), QTcF >500 ms in 5.6% and mean QTcF change from baseline >60 ms in 9.3% of patients.	Dose-dependent changes less significant in 20 mg/dose, 30 mg/dose, or 40 mg/dose	[9]
Vorinostat (Zolinza) A single supratherapeutic dose of 800 mg. EKG was monitored before treatment and for 24 h post-dose.	Among 25 patients with advanced-stage cancer aged 29–78 years old, placebo-adjusted mean QTcF change from baseline <10 ms at every time point, one patient >450 ms, none >480 ms.	800 mg of vorinostat at a single dose was generally tolerated	[12]
Vorinostat (Zolinza)	QT prolongation: In 3.5–6.0% of cases in a report by Asteggiano et al., 2021, case details unknown.	A greater risk of torsade de pointes in females/older patients	[10]
Belinostat increasing doses as a continuous intravenous infusion over 48 h with chemotherapy, maximum tolerated dose of 1000 mg/m^2^	QT prolongation: Among 26 patients with thymic epithelial tumors aged 23–76 years old, 9 (35%) had QTc prolongation, 1 event >500 ms, 2 had electrolyte abnormalities and dehydration.	Cases of electrolyte abnormalities	[11]

QTcF: QT interval corrected for heart rate using Fridericia’s formula.

**Table 2:** HDAC3 depletion alters the expression of genes related to genetic long QT (LQT) syndromes.

LQT#	Gene	Full Name	Syndrome	Fold Change (KO vs. WT)	q-Value
LQT1	KCNQ1	potassium voltage-gated channel, subfamily Q, member 1	Romano–Ward syndrome, Jervell and Lange-Nielsen syndrome	−3.2	2.9 × 10^−58^
LQT2	KCNH2	potassium voltage-gated channel, subfamily H, member 2	Romano–Ward syndrome	−3.7	2.2 × 10^−117^
LQT3	SCN5A	sodium channel, voltage-gated, type V, alpha	Romano–Ward syndrome	−2.7	4.1 × 10^−182^
LQT4	ANK2	ankyrin 2	Romano–Ward syndrome	−1.4	1.3 × 10^−11^
LQT5	KCNE1	potassium voltage-gated channel, Isk-related subfamily, member 1	Romano–Ward syndrome, Jervell and Lange-Nielsen syndrome	−12.4	8.6 × 10^−19^
LQT6	KCNE2	potassium voltage-gated channel, Isk-related subfamily, gene 2	Romano–Ward syndrome	−1.2	9.4 × 10^−01^
LQT7	KCNJ2	potassium inwardly rectifying channel, subfamily J, member 2	Andersen syndrome	−1.5	8.2 × 10^−15^
LQT8	CACNA1C	calcium channel, voltage-dependent, L type, alpha 1C subunit	Timothy syndrome	−1.5	3.4 × 10^−36^
LQT9	CAV3	caveolin 3	Romano–Ward syndrome	−1.9	8.5 × 10^−26^
LQT10	SCN4B	sodium channel, type IV, beta	Romano–Ward syndrome	−2.5	9.3 × 10^−94^
LQT11	AKAP9	A kinase (PRKA) anchor protein 9	Romano–Ward syndrome	−1.3	2.3 × 10^−5^
LQT12	SNTA1	syntrophin, acidic 1	Romano–Ward syndrome	1.0	8.4 × 10^−1^
LQT13	KCNJ5	potassium inwardly rectifying channel, subfamily J, member 5	Romano–Ward syndrome	−3.9	7.6 × 10^−98^

## Data Availability

The RNA-seq dataset analyzed in this study is publicly available in the Gene Expression Omnibus (GEO) under accession number GSE249901.

## Supplementary Materials

The following supporting information can be downloaded at: https://www.mdpi.com/article/10.3390/cells15100902/s1, Figure S1: Expression of cardiac KCN genes in mice depleted of cardiac HDAC3; Figure S2: EKG tracings in MCM mice injected with tamoxifen; Figure S3: Expression of cardiac HDAC genes in mice administered with romidepsin; Figure S4: Expression of cardiac HDAC genes in mice administered with mocetinostat.
