# Supplementary material for: Cardiac HDAC3 Disruption Contributes to HDAC Inhibitor-Induced QT Prolongation
Source: Cells. 2026 May 14;15(10):902. doi: 10.3390/cells15100902 (PMC13204737; doi:10.3390/cells15100902)
Supplement: Supplementary file 1 [file cells-15-00902-s001.zip › cells-4104446-supplementary.pdf]

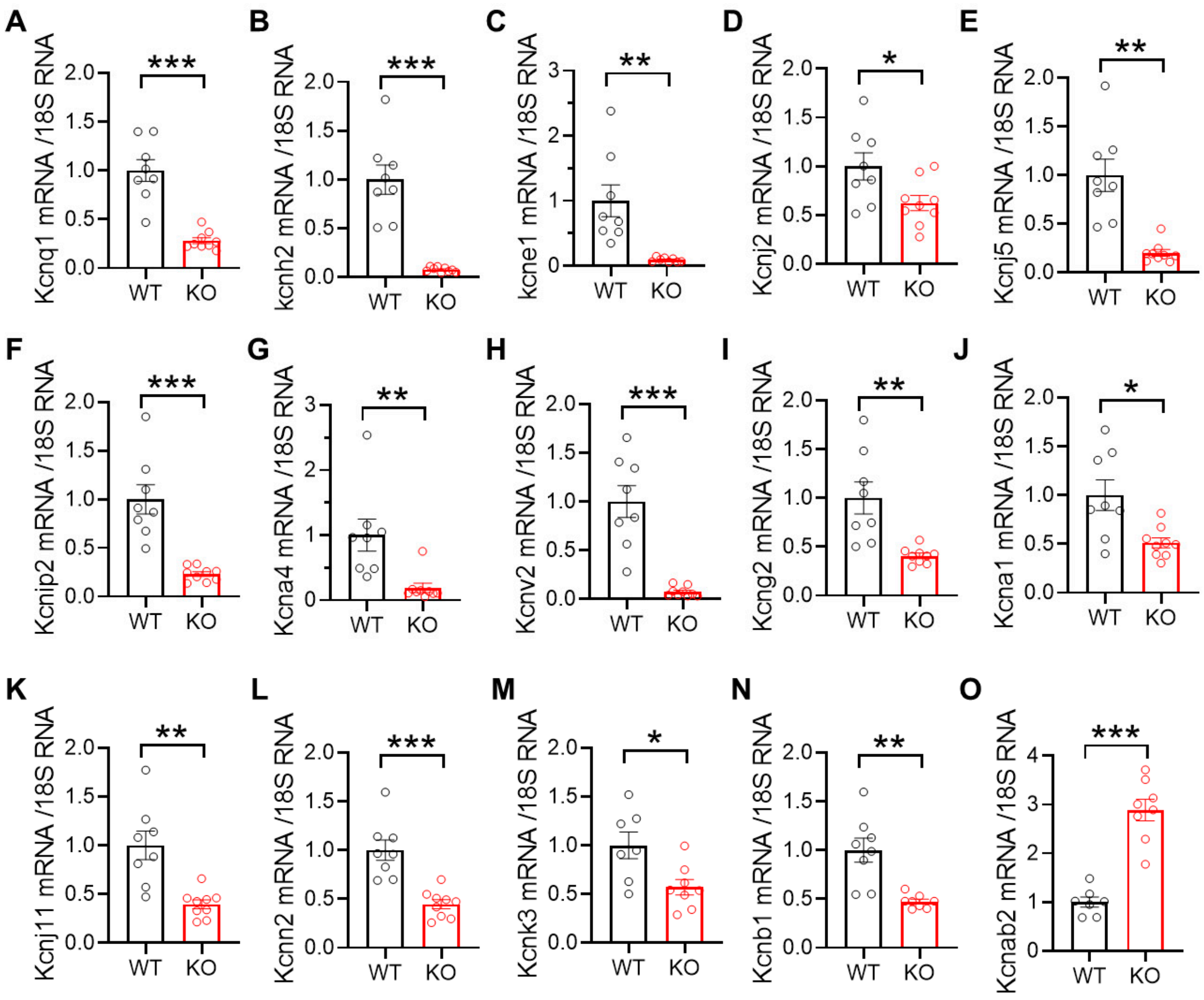

Supplementary Materials Figure S1. Expression of cardiac KCN genes in mice depleted of cardiac HDAC3
A-O, Quantitative PCR analysis of cardiac mRNA expression for the indicated genes in WT and KO mice, n=7-9 per group. mRNA levels were normalized to 18S rRNA. Data are mean ± SEM. *P<0.05, **P<0.01, ***P<0.001. Data were obtained from combined female and male mice aged 2–6 months. WT, HDAC3$^{fl/fl}$ mouse. KO, HDAC3$^{fl/fl}$:MCK-Cre mouse.

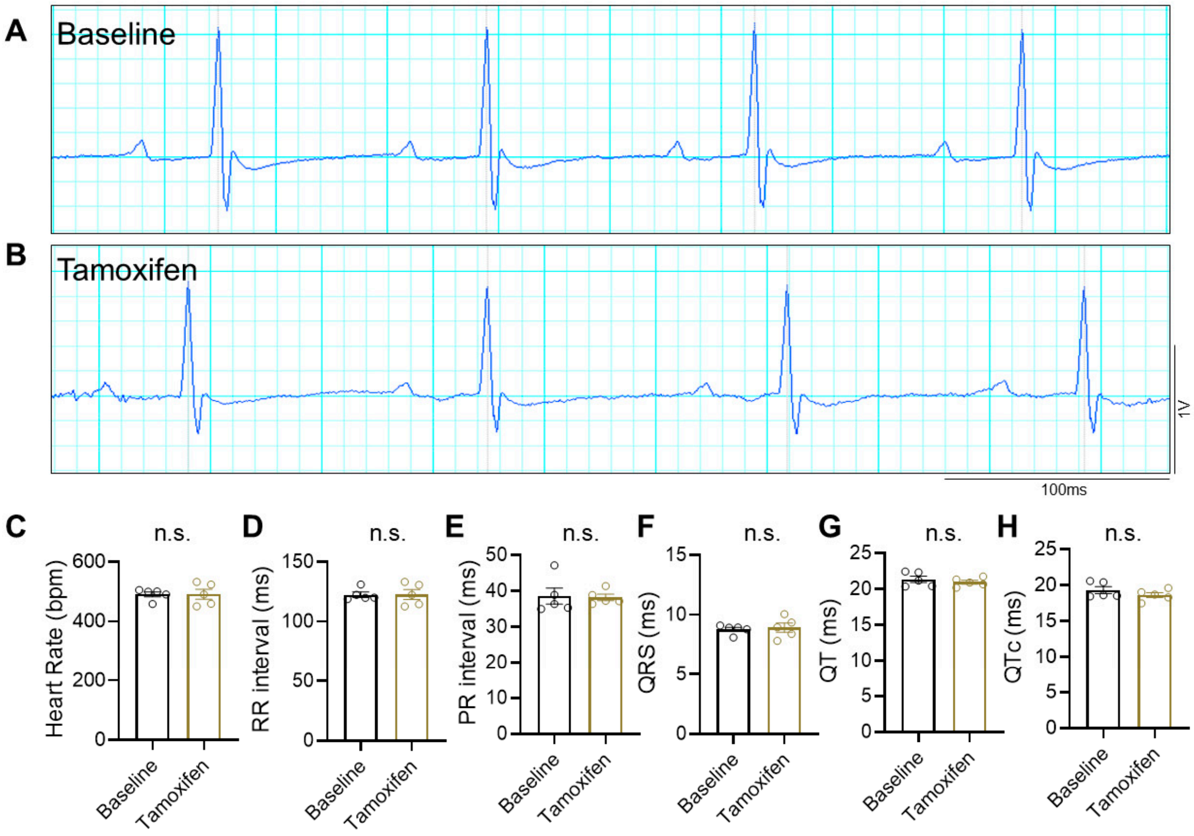

Supplementary Materials Figure S2. EKG tracings in MCM mice injected with tamoxifen

A-B, Representative image of surface EKG tracings of a MCM mouse before and after tamoxifen administration. **C,** Heart rate during surface EKG recording, n=5 per group. **D,** RR interval n=5 per group. **E,** PR interval. n=5 per group. **F,** QRS duration, n=5 per group. **G,** QT duration, n=5 per group. **H,** QTc duration, n=5 per group. MCM mice were injected with tamoxifen 20 mg/kg per day for 4 consecutive days. MCM, Mer-Cre-Mer mouse. n.s. indicates not significant. Data were obtained from combined female and male mice aged 2–4 months.

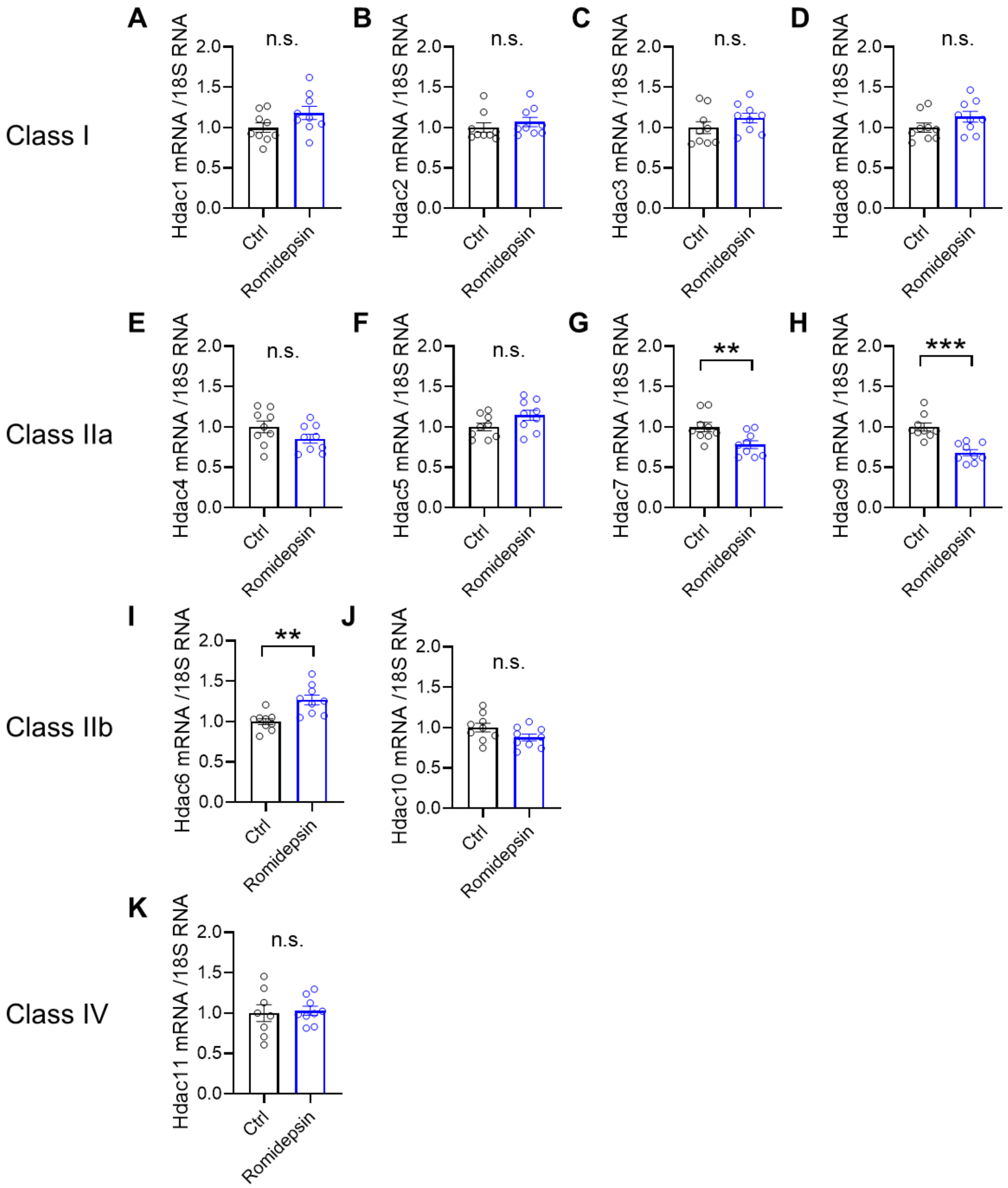

Supplementary Materials Figure S3. Expression of cardiac HDAC genes in mice administered with romidepsin

A-K, Quantitative PCR analysis of cardiac mRNA expression for the indicated genes in wild-type C57BL/6 mice treated with vehicle (Ctrl) or romidepsin (1 mg/kg, intraperitoneally), n=8-9 per group. **A-D**, Class I HDACs. **E-H**, Class IIa HDACs. **I-J**, Class IIb HDACs. **K**, Class IV HDAC. Mouse hearts were harvested 6 hours after romidepsin injection. mRNA levels were normalized to 18S rRNA. Data are mean ± SEM. *P<0.05, **P<0.01, ***P<0.001, n.s. indicates not significant. Data were obtained from combined female and male mice aged 2–4 months.

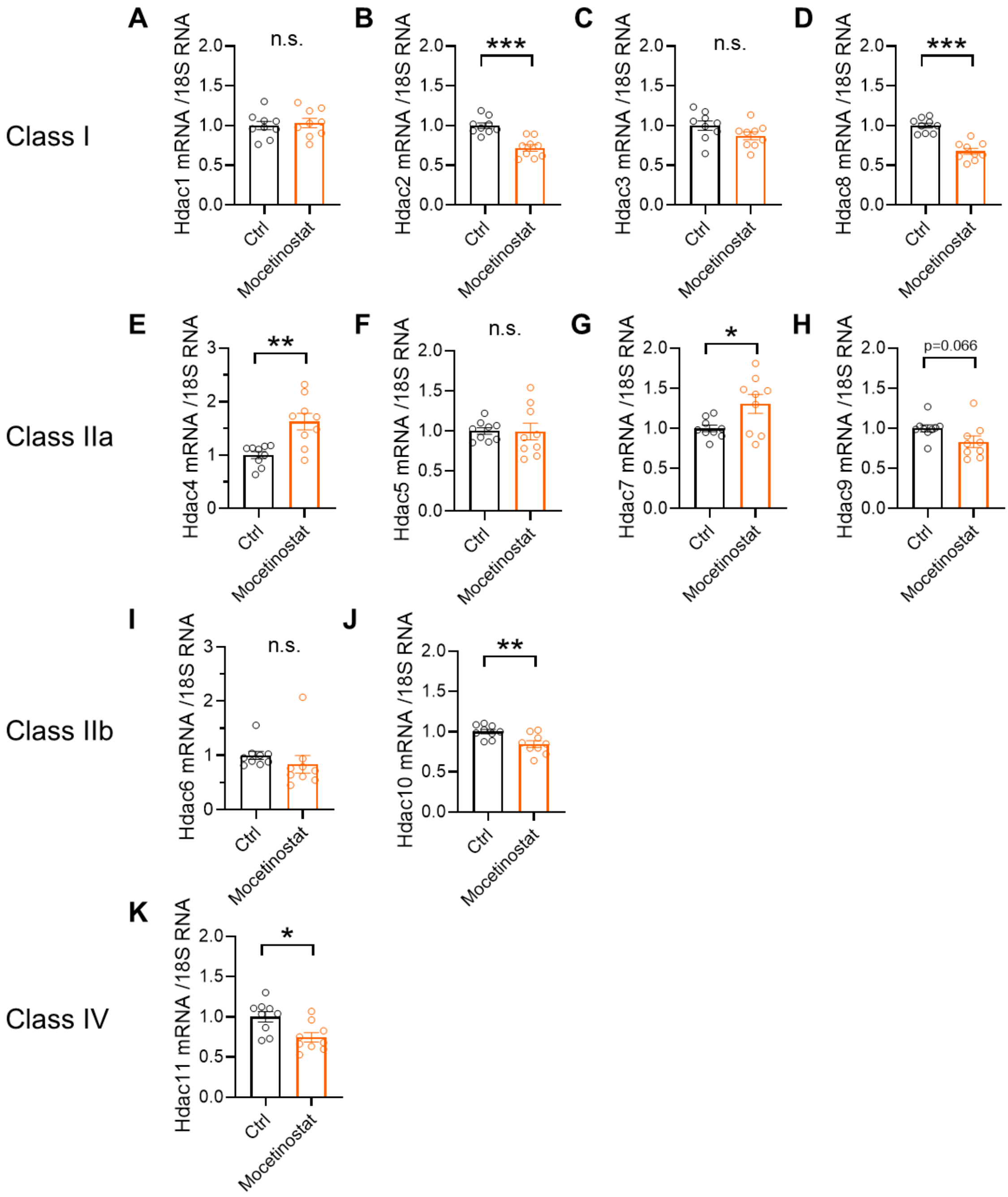

Supplementary Materials Figure S4. Expression of cardiac HDAC genes in mice administered with mocetinostat

A-K, Quantitative PCR analysis of cardiac mRNA expression for the indicated genes in wild-type C57BL/6 mice treated with vehicle (Ctrl) or mocetinostat (10 mg/kg, intraperitoneally), n=9 per group. **A-D**, Class I HDACs. **E-H**, Class IIa HDACs. **I-J**, Class IIb HDACs. **K**, Class IV HDAC. Mouse hearts were harvested 6 hours after mocetinostat injection. mRNA levels were normalized to 18S rRNA. Data are mean ± SEM. *P<0.05, **P<0.01, ***P<0.001, n.s. indicates not significant. Data were obtained from combined female and male mice aged 2–4 months.
